# Determination of Temperature-Dependent Elastic Constants of Steel *AISI 4140* by Use of In Situ X-ray Dilatometry Experiments

**DOI:** 10.3390/ma13102378

**Published:** 2020-05-21

**Authors:** Dominik Kiefer, Jens Gibmeier, Andreas Stark

**Affiliations:** 1 Institute for Applied Materials (IAM-WK), Karlsruhe Insitute of Technology (KIT), Engelbert-Arnold-Str. 4, 76131 Karlsruhe, Germany; jens.gibmeier@kit.edu; 2 Institute of Materials Research, Helmholtz-Zentrum Geesthacht (HZG), Max-Planck-Str. 1, 21502 Geesthacht, Germany; andreas.stark@hzg.de

**Keywords:** in situ X-ray diffraction, elastic constants, synchrotron radiation, tempering steel, AISI 4140, dilatometry

## Abstract

In situ dilatometry experiments using high energy synchrotron X-ray diffraction in transmission mode were carried out at the high energy material science beamline P07@PETRAIII at DESY (Deutsches Elektronen Synchrotron) for the tempering steel AISI 4140 at defined mechanical loading. The focus of this study was on the initial tempering state (ferrite) and the hardened state (martensite). Lattice strains were calculated from the 2D diffraction data for different hkl planes and from those temperature-dependent lattice plane specific diffraction elastic constants (DECs) were determined. The resulting coupling terms allow for precise stress analysis for typical hypoeutectoid steels using diffraction data during heat treatment processes, that is, for in situ diffraction studies during thermal exposure. In addition, by averaging hkl specific $Young^{\prime }s\;moduli$ and Poissonratios macroscopic temperature-dependent elastic constants were determined. In conclusion a novel approach for the determination of phase-specific temperature-dependent DECs was suggested using diffraction based dilatometry that provides more reliable data in comparison to conventional experimental procedures. Moreover, the averaging of lattice plane specific results from in situ diffraction analysis supply robust temperature-dependent macroscopic elastic constants for martensite and ferrite as input data for heat treatment process simulations.

## 1. Introduction

Manufacturing of technical components is always accompanied by the generation of characteristic residual stress distributions. Oftentimes compressive residual stresses, particularly in the near surface regions, are desired, since in most applications the integrity of the parts can be effectively enhanced as, for example, fatigue strength and wear resistance [1,2]. These residual stress states are specifically induced through the manufacturing process or by means of post (heat) treatments. Knowledge of the residual stress states of components and hence their exact determination is of great importance for mechanical engineering, especially for components design and dimensioning. In the course of steady improvement of analysis methods an increasing amount of studies focuses on in situ determination of the development of mechanical or thermal stresses during different kinds of processing. Hence, there is an increasing demand for the provision of data evaluation procedures for proper handling of large data volumes and of qualified evaluation parameters, for example, temperature-dependent elastic constants. Since in many cases, thermal processes are accompanied by phase transformations, X-ray or neutron diffraction analysis is the method of choice for the determination of phase specific diffraction data that allows for monitoring both, phase transformations and the development of local phase specific residual stresses. In polycrystalline materials stress analysis by means of X-ray diffraction always requires coupling constants [3] to convert lattice strains into mechanical stresses. For the assumption of quasi-isotropic specimens hkl specific diffraction elastic constants (DECs) [4] can be calculated from single crystal elastic constants from the stiffness tensor $C_{ij}$ or the corresponding compliance tensor $S_{ij}$. For this different model approaches are used. These approaches, for example, Eshelby-Kröner model [5,6], describe the coupling of crystallites in the polycrystalline compound. For room temperature applications the single crystal elastic constants are tabulated for most elements, many intermetallics and ceramic compounds in databases like Landolt-Börnstein [7]. For individual cases values for $C_{ij}$ at elevated temperatures *T* are given or a so called temperature factor $T_{ij}$ allows for the calculation of $C_{ij}\left(T\right)$, assuming a linear dependency on *T* [7]. Apart from this, it is well-known that the (macroscopic) elastic behaviour of materials changes with increasing temperature. In literature it is shown that this trend is not entirely linear [8,9]. These deviations from linearity in the elastic behaviour may cause significant errors in the determination of stresses at elevated temperatures using DECs based on room temperature single crystal constants. To improve the reliability and validity of high temperature stress analysis, as for example, in our own work about in situ laser surface hardening [10,11] there is the necessity of determining high temperature DECs for proper data evaluation. Since the temperature dependency of diffraction elastic constants for specific hkl lattice planes does not necessarily have to correspond with that one of the macroscopic elastic constants, a hkl specific consideration is essential. In case where no reliable DECs are accessible, a possible workaround is the elaborate experimental determination e.g., according to ASTM1426-14 [12] in which X-ray stress analysis on the basis of the well-known $\sin^{2}\psi$-method [13] is carried out during mechanical loading. Here, we report about a more elegant approach for the proper and reliable determination of polycrystal diffraction elastic constants at elevated temperatures for quasi-isotropic materials by using in situ X-ray diffraction load dilatometry experiments in combination with high energy synchrotron X-rays. The motivation of this work is based on the necessity of reliable DECs for the accurate determination of stresses using our well-established measurement setup for in situ stress analysis during laser surface hardening [10,11]. In this regard the DECs determined using the herein proposed dilatometry approach will be used to improve current experimental data on laser surface hardening. Furthermore, averaged macroscopic elastic parameters can directly be used to improve finite element (FE) heat treatment process simulations [14].

## 2. Materials and Methods

### 2.1. Experimental Procedure

The investigated material is the common tempering steel AISI 4140 in a quenched and tempered state. Cylindrical samples (full cylinders) with the dimension Ø4 mm×10 mm were mechanically prepared and additionally tempered at $510\;{}^{\circ }C$ for 90 min in inert gas atmosphere (Ar) to relieve residual stresses and provide a nearly stress free sample state. The chemical composition is shown in Table 1.

**Figure 1:**
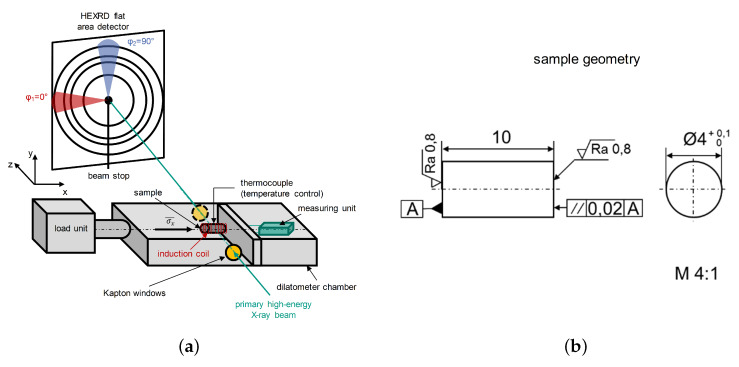
(**a**) Scheme of the experimental setup and (**b**) technical drawing of the sample geometry.

At the high energy material science beamline P07@PETRA III at DESY (Deutsches Elektronen Synchrotron) in Hamburg, Germany, operated by the Helmholtz-Zentzrum Geesthacht (HZG), multiple in situ X-ray load dilatometry experiments were performed. A double crystal monochromator (DCM) equipped with two Si(111) Laue crystals provide a high flux X-ray beam with an energy of E=100 keV (λ=0.124 Å). A cross slit aperture was used to shape the beam cross section to a square of 0.6 mm×0.6 mm. Debye-Scherrer rings were recorded with a XRD flat area detector Perkin Elmer XRD1621 (2048×2048 pixels). The chosen sample-detector distance was about 1460 mm and the frame rate was about 0.3Hz. A quenching dilatometer of type DIL 805A/D with a load unit from the company TA Instruments equipped with Kapton^®^ windows that allow the primary X-ray beam to enter and the diffracted beam to exit the dilatometer chamber was used. A scheme of the experimental setup is given in Figure 1a and a technical drawing of the sample geometry in Figure 1b.

**Figure 2:**
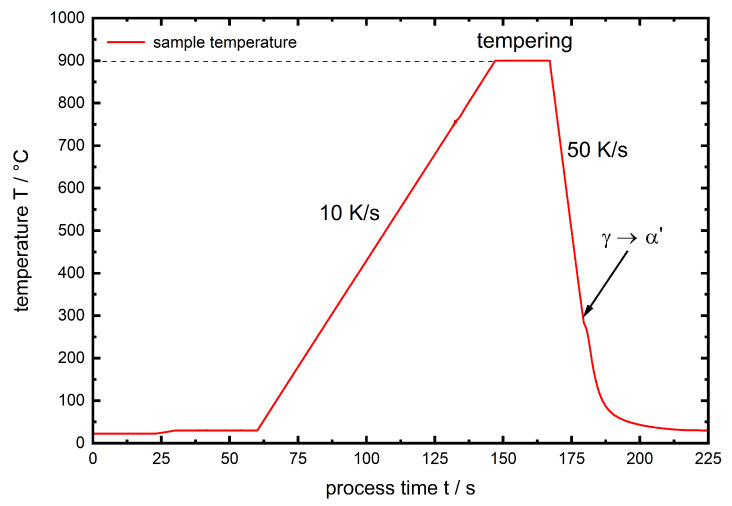
Temperature course of the pre-heat treatment for the martensite experiments.

The performed experiments are divided by phase into ferrite, describing the initial high tempered sample state of AISI 4140 and martensite for which the samples were heated up to $900\;{}^{\circ }C$, tempered for 60 s and quenched by a He gas stream in the dilatometer with a cooling rate of $50\;Ks^{-1}$. To illustrate the link to Table 2 the phase assignments are written italic. In Figure 2 the temperature course of the described martensite heat treatment is given. The kink observed during quenching can clearly be assigned to martensitic transformation.

The samples were heated up with a heating rate of $10\;Ks^{-1}$ to the specified test temperature by thermocouple control. Then force-controlled axial load experiments were carried out at different temperatures (isothermal experiment control) for both body centered phases (ferrite&martensite). Each single load step was held for at least 15 s. For each temperature 4 lattice planes hkl were investigated and the plane specific temperature-dependent elastic constants and hence the corresponding DECs were determined. The analyzed lattice planes of the body centered α-iron solid solutions were {110}, {200}, {211} and {220}. The complete experimental plan is given in Table 2 together with the applied maximum stress that was considered for evaluation.

Furthermore, the datasets obtained for the different lattice planes were used to average values to also provide values for the macroscopic temperature-dependent elastic constants $\bar{E}$ and $\bar{\nu }$.

### 2.2. Data Evaluation

The Debye Scherrer rings that were recorded time resolved were evaluated according to Reference [15] in the two principal directions $\varphi_{1}=0{}^{\circ }\left(x\right)$ and $\varphi_{2}=90{}^{\circ }\left(y\right)$, see also Figure 1. For this purpose pie slices with an azimuthal range of $10{}^{\circ }\left(\pm 5{}^{\circ }\right)$ were defined to increase statistics. Thus, for each time step one dimensional plots of intensity against 2θ were obtained and further analyzed using MATLAB^TM^. For the determination of peak positions the different diffraction lines were fitted using a Pseudo-Voigt function. At the investigated temperatures the plane specific lattice spacings $d^{hkl}$ and based on those the strains were calculated according to Bragg’s law in both directions. According to Hooke’s law for isotropic materials the stress under uniaxial compression in load direction ${\bar{\sigma }}_{x}$ is given by Equation (1):

(1) $${\bar{\sigma }}_{x}=\bar{E}\;\cdot \;{\bar{\varepsilon }}_{x},$$

where $\bar{E}$ is the macroscopic $Young^{\prime }s\;modulus$ and ${\bar{\varepsilon }}_{x}$ is the principal strain in load direction *x*. Subsequently, we assume the macroscopic stress ${\bar{\sigma }}_{x}$ is equal to the plane specific stress $\sigma_{0^{\circ }}^{hkl}$. For the cylindrical samples the strain components perpendicular to the *x* direction are axisymmetric. Hence, the principle strain directions *x*, *y* and *z* can be substituted in agreement with Figure 1 according to Equations (2) and (3):

(2) $$x=(\varphi_{1}=0^{\circ })\;,$$

(3) $$y=z=(\varphi_{2}=90^{\circ })\;.$$

This leads to a simple expression for the lattice plane-specific axial strain (Equation (4)):

(4) $$\begin{array}{c} \varepsilon_{0^{\circ }}^{hkl}=\frac{1}{E^{hkl}}\;\cdot \;\sigma_{0^{\circ }}^{hkl}, \end{array}$$

and the hkl-specific lateral contraction strain $\varepsilon_{90^{\circ }}^{hkl}$ is given by (Equation (5))

(5) $$\varepsilon_{90^{\circ }}^{hkl}=-\nu^{hkl}\;\cdot \;\varepsilon_{0^{\circ }}^{hkl}.$$

Here $\nu^{hkl}$ is the hkl-specific Poissonratio. Both Equations (4) and (5) express linear terms with slopes of $E^{hkl^{-1}}$ and $\nu^{hkl}$ respectively. Summarizing the single lattice plane specific data allows for the calculation of macroscopic elastic constants. Daymond [16] presented different approaches for averaging strain data evaluated from multiple diffraction peaks hkl to determine a continuum mechanics equivalent strain (MethodA). The macroscopic mean strain ${\bar{\varepsilon }}_{x}$ is herein averaged from hkl specific data as in Equation (6)

(6) $${\bar{\varepsilon }}_{x}=\frac{\sum_{n_{hkl}}^{N}T^{hkl}m^{hkl}E^{hkl}\;\cdot \;\varepsilon_{0^{\circ }}^{hkl}}{\sum_{n_{hkl}}^{N}T^{hkl}m^{hkl}E^{hkl}},$$

where $T^{hkl}$ is the texture index [17] which was set equal to 1 in first approximation for all hkl, under the assumption of a texture free polycrystal, $m^{hkl}$ is the peak multiplicity and $E^{hkl}$ are the plane specific $Young^{\prime }s\;moduli$. The macroscopic $Young^{\prime }s\;modulus$ $\bar{E}$ can now be calculated on the basis of hkl specific elastic constants by insertion of Equation (6) into Equation (1). In this paper we set up a similar equation to calculate the macroscopic lateral contraction strain ${\bar{\varepsilon }}_{y,z}$ using the lattice plane specific Poissonratios$\nu^{hkl}$, Equation (7).

(7) $${\bar{\varepsilon }}_{y,z}=\frac{\sum_{n_{hkl}}^{N}T^{hkl}m^{hkl}\nu^{hkl}\;\cdot \;\varepsilon_{90^{\circ }}^{hkl}}{\sum_{n_{hkl}}^{N}T^{hkl}m^{hkl}\nu^{hkl}}.$$

The results of Equations (6) and (7) are used to calculate the macroscopic $Poisson\;ratio\;\bar{\nu }$ according to Equation (8).

(8) $${\bar{\varepsilon }}_{y,z}=-\bar{\nu }\;\cdot \;{\bar{\varepsilon }}_{x}.$$

The relationships between hkl specific elastic constants and the DECs$s_{1}^{hkl}$ and $\frac{1}{2}s_{2}^{hkl}$ are given, according to Reference [4], in Equation (9):

(9) $$s_{1}^{hkl}=\frac{-\nu^{hkl}}{E^{hkl}}\;,\;\frac{1}{2}s_{2}^{hkl}=\frac{-\nu^{hkl}+1}{E^{hkl}}.$$

Errorbars for $E^{hkl}$ and $\nu^{hkl}$, respectively $\bar{E}$ and $\bar{\nu }$, are based on the quality of linear regression (standard deviation) and propagated in the calculation of DECs.

## 3. Results and Discussion

### 3.1. hkl Specific Elastic Constants

**Figure 3:**
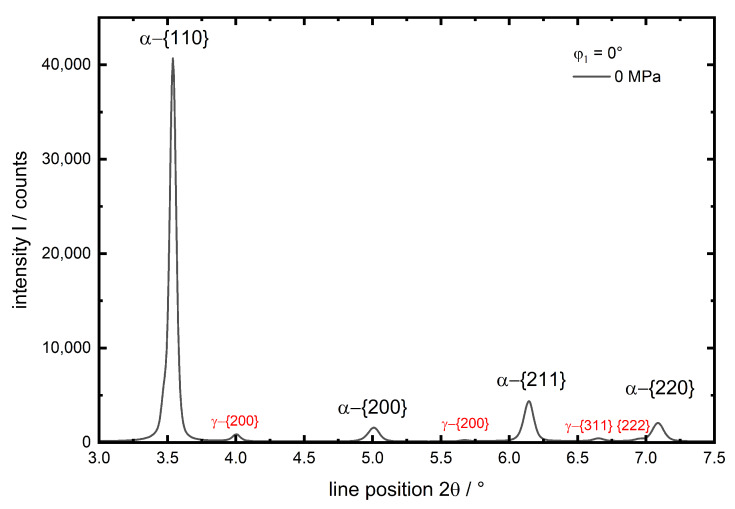
Diffractogram from experiment no. 1 ($martensite,\;30\;{}^{\circ }C$) before loading in $x\;(\varphi_{1}=0{}^{\circ })$ direction. Observed lattice planes hkl are indexed.

The different experiments were numbered from no. 1 to 7 according to the applied temperatures between $30\;{}^{\circ }C$ and $600\;{}^{\circ }C$ (see Table 2). In Figure 3 a diffractogram for $\varphi_{1}=0{}^{\circ }$ and 0 MPa is given for experiment no. 1 ($martensite,\;T=30\;{}^{\circ }C$) exemplarily.

**Figure 4:**
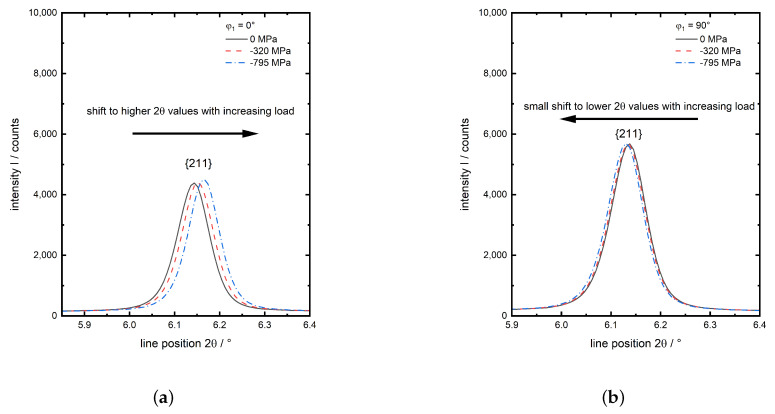
Detailed graphs of the {211} α-Fe from experiment no. 1 ($martensite,\;30\;{}^{\circ }C)$ for three load steps, (**a**) in load direction $x\;(\varphi_{1}=0{}^{\circ })$ and (**b**) in transverse direction $y\;(\varphi_{2}=90{}^{\circ })$.

The four investigated diffraction planes hkl of the α-iron phase are indexed. The much smaller γ peaks are indexed red. These are only observed for experiment no. 1 and 2. They belong to retained austenite after quenching and are neither existent for the initial (ferrite) state nor the investigations of martensite at elevated temperatures, since the intensity of the γ peaks and hence the amount of retained austenite, decreases at elevated temperatures (experiments 4 and 6). Consequently the amount of retained austenite is very small (below 3%, experiment no. 1), which is no amount for significant load partitioning in comparison to the martensite, this phase is not taken into account during data evaluation. A more detailed, representative view on the behaviour of the {211} peak with increasing load for experiment no. 1 in both evaluated directions *x* and *y* is given in Figure 4. In load direction *x* the peak shows a clear shift to higher 2θ values with increasing compressive load (Figure 4a), whereas in the transverse direction *y* the less pronounced opposite is observed (Figure 4b) due to the much lower transverse strain compared to the strain caused by axial compression.

**Figure 5:**
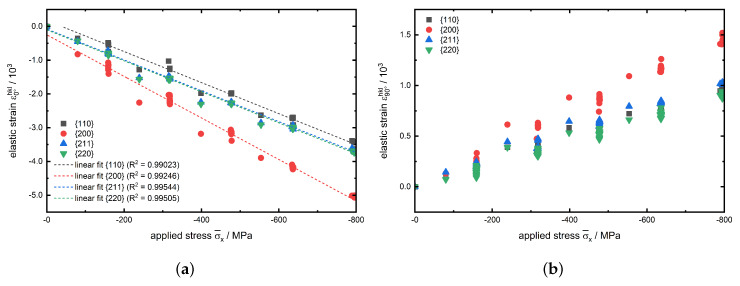
(**a**) Axial strain $\varepsilon_{0^{\circ }}^{hkl}$ in load direction against the applied stress ${\bar{\sigma }}_{x}$ plus the linear fit interpolation as dashed lines and (**b**) lateral strain $\varepsilon_{90^{\circ }}^{hkl}$ against the applied stress ${\bar{\sigma }}_{x}$ for the investigated lattice planes hkl of experiment no. 1.

In Figure 5a the hkl specific determined strain in load direction $x\;(\varphi_{1}=0{}^{\circ })$ is exemplarily plotted against the applied compressive stress ${\bar{\sigma }}_{x}$ for experiment no. 1. Whereas in Figure 5b the corresponding course of transverse strain $\varepsilon_{90^{\circ }}^{hkl}$ is shown. The lattice plane specific strains are calculated from the peak positions 2θ according to Equation (10):

(10) $${\bar{\varepsilon }}^{hkl}=-0.5\;\cdot \;\cot\left(\theta_{0}^{hkl}\right)\left(2\theta^{hkl}-2\theta_{0}^{hkl}\right),\;$$

where $2\theta_{0}^{hkl}$ is the line position determined after reaching the test temperature before mechanical loading.

**Figure 6:**
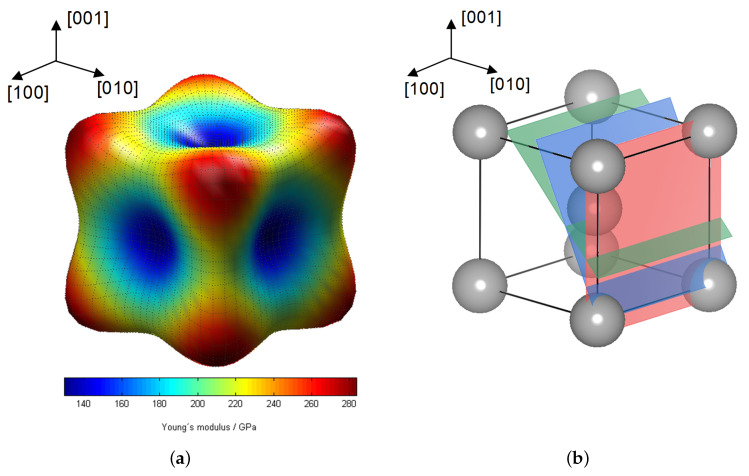
(**a**) Elastic anisotropy of the α-Fe unit cell at 300 K (calculated with DECcalc [18]) and (**b**) scheme of the α-iron unit cell with illustration of the investigated lattice planes.

Furthermore in Figure 5a, the associated linear fits are shown as dashed lines for the investigated lattice planes hkl. According to Equation (4) the slopes of these distributions correspond to the inverse $Young^{\prime }s\;moduli\;{E^{hkl}}^{-1}$ for the specific lattice plane of type hkl. As expected, the slopes of the linear fits for the {110} and {220} reflections are nearly identical, since they represent the same lattice plane family. Obviously, the determined $Young^{\prime }s\;modulus\;E^{hkl}$ for the {211} lattice planes nearly coincides with the value for the {110} and {220} planes with a value of approx. 218GPa. It is observed that the linear fit for {200} has a much lower slope resulting in a significant lower value for $E^{hkl}$ with about 167GPa. These results are in good agreement with literature and are explained by the elastic anisotropy of the α-iron unit cell, cf. Figure 6, which can mathematically be expressed by the elastic anisotropy factor Γ. In case of cubic crystals, Γ is given as:

(11) $$\Gamma_{hkl}=\frac{h^{2}k^{2}+k^{2}l^{2}+l^{2}h^{2}}{{\left(h^{2}+k^{2}+l^{2}\right)}^{2}}.$$

In Figure 6b the locations of the herein investigated lattice planes hkl in a body-centered cubic (bcc) unit cell is shown. Since here $\Gamma_{200}<\Gamma_{110}=\Gamma_{220}=\Gamma_{211}$ is valid here the {200} lattice plane has to be the least stiff plane with the lowest $Young^{\prime }s\;modulus$. This fact is confirmed by Figure 5a where the {200} linear fit has the smallest slope. This correlation is illustrated in Figure 6a, showing the elastic anisotropy of the $Young^{\prime }s\;modulus$ of the bcc α-Fe unit cell.

**Figure 7:**
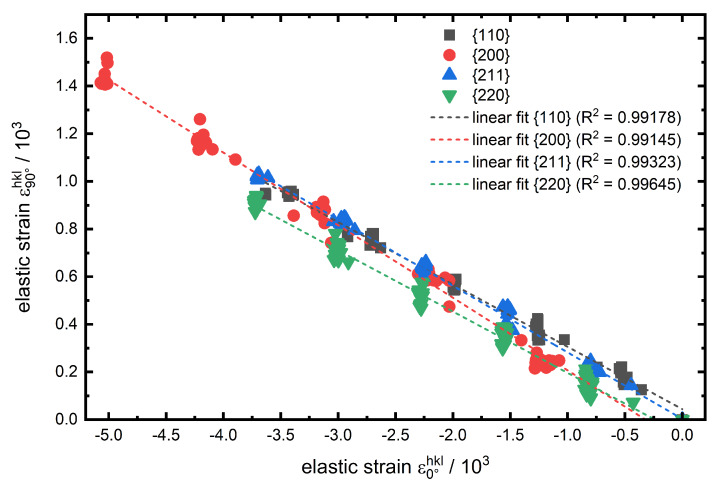
Transverse elastic strain $\varepsilon_{90^{\circ }}^{hkl}$ against axial elastic strain $\varepsilon_{0^{\circ }}^{hkl}$ and the linear fit interpolation for the investigated lattice planes hkl of experiment no. 1.

For the determination of the lattice plane specific Poissonratio $\nu^{hkl}$ a plot of the transverse strain $\varepsilon_{90^{\circ }hkl}$ against the axial strain $\varepsilon_{0^{\circ }hkl}$ is shown for experiment no. 1 in Figure 7. The determined slopes from the linear fits correspond (according to Equation (5)) to $-\nu^{hkl}$. Here again, the absolute values of the slopes for {110} and {220} are nearly identical at about 0.26. The {211} lattice plane has a slightly higher Poissonratio of about 0.28. An even higher value of about 0.3 is determined for the low-indexed {200} lattice plane, which again can also be attributed to the elastic anisotropy of the unit cell and the much higher transverse strain (at equal maximum load) in Figure 7 also gives a first indication for the higher values for $\nu^{hkl}$.

**Figure 8:**
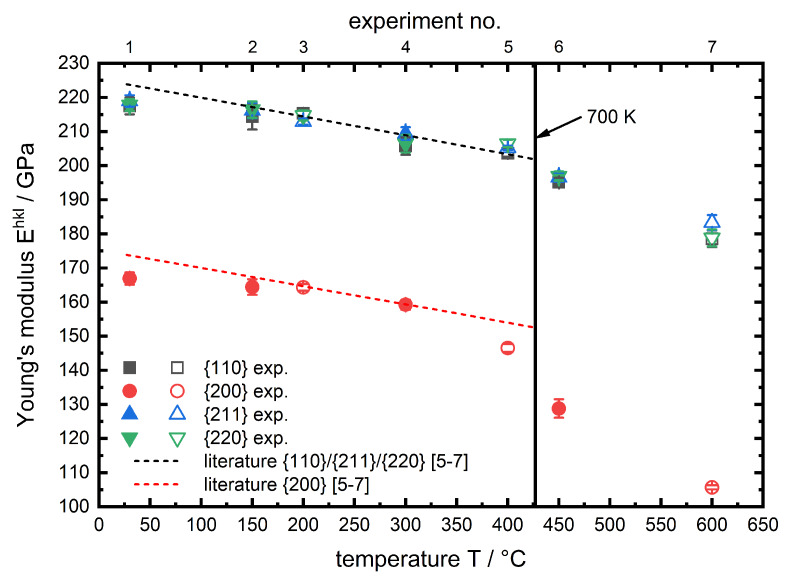
Temperature-dependent hkl specific $Young^{\prime }s\;moduli$ for the investigated phases of AISI 4140. Literature data shown as a 1st order polynomial fit based on single crystal constants from Reference [7] and calculated according to Kröner [6]. Filled symbols for martensite and blank symbols for ferrite phase.

In Figure 8 the hkl specific $Young^{\prime }s\;moduli$ $E^{hkl}$ for all experiments are plotted against the temperature.

**Figure 9:**
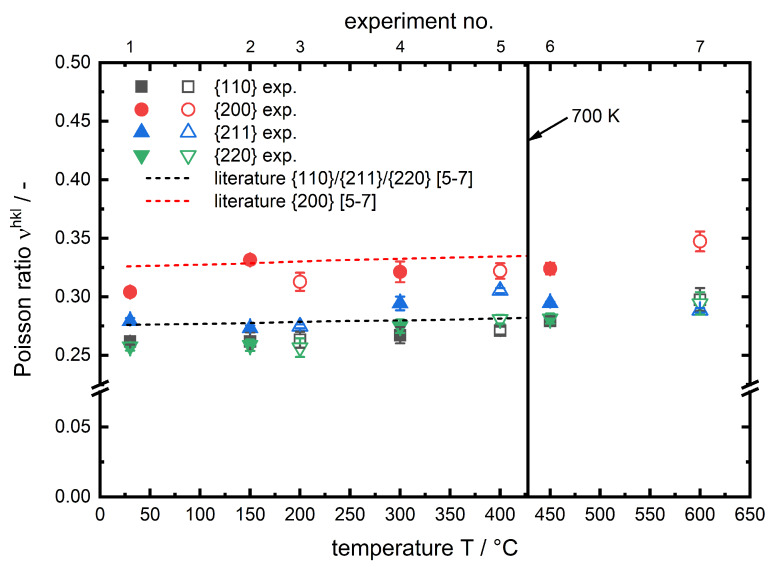
Temperature-dependent hkl specific Poissonratios for the investigated phases of AISI 4140. Literature data shown as a 1st order polynomial fit based on single crystal constants from Reference [7] and calculated according to Kröner [6]. Filled symbols for martensite and blank symbols for ferrite phase.

The corresponding plot for the Poissonratio $\nu^{hkl}$ is shown in Figure 9.

In both figures the results are plotted alongside data based on single crystal elastic constants from References [6,7] for α-iron in the range of 27 to $427\;{}^{\circ }C$. Following the temperature course of hkl specific $Young^{\prime }s\;modulus$ and Poissonratio one can observe that there is no distinct difference between the two investigated phases (ferrite,martensite). Therefore the reason can be seen in the structure of the two phases. Ferrite has a body centered cubic (bcc) lattice structure, while the lattice structure of martensite is tetragonal body centered (tbc). Due to the low carbon content the tetragonal distortion of martensite is rather low (≈0.2%) [19] and therefore the lattice structure is close to cubic, which explains that the micromechanical behaviour of ferrite and martensite for the hypoeutectoid steel in the investigated temperature range is almost identical to one another, hence for both phases a similar trend can be observed. The hkl specific $Young^{\prime }s\;moduli$ decrease with increasing temperature in all cases. This behaviour is well known in literature for macroscopic $Young^{\prime }s\;moduli$ [8]. It can be explained using the binding potential. With increasing temperature the atoms start vibrating more, which results in an increase of the average interatomic equilibrium distance $r_{eq}$ (thermal strain). Since the $Young^{\prime }s\;modulus$ can be described as the 2nd derivative of the binding potential at $r_{eq}\left(T\right)$ it has to decrease with increasing temperature. The interatomic equilibrium distance $r_{eq}$ is much higher for the {200} lattice planes compared to other investigated lattice planes. In both figures the dashed lines show the corresponding lattice plane specific results, based on single crystal constants from Reference [7], which are calculated according to Kröner [6] up to a temperature of $427\;{}^{\circ }C$. In this temperature range the results of the in situ X-ray load dilatometry studies are in very good agreement with the literature data for pure α-iron. A further temperature rise leads to a steeper decrease of $E^{hkl}$, in particular for the {200} lattice plane. This behaviour for steels is neither observed nor discussed (specifically for steel) in literature. But for macroscopic $Young^{\prime }s\;moduli$ of oxide ceramics, Watchman et al. [8] described the non-linear decrease of the elastic constant approach, for a wide temperature range, using a exponential function. Li et al. [9] observed a comparable temperature course for the macroscopic $Young^{\prime }s\;modulus$ of hafnium carbide HfC at very high temperatures and expanded the exponential approach analytically. Regarding the presented results for ferritic steels we expect similar correlations causing this effect. However, in lists of tables i. a. Landolt-Börnstein [7] for pure iron there is unfortunately no temperature factor given for $C_{11}$ for temperatures higher than 700 K ($427\;{}^{\circ }C$). This does not allow for a reliable calculation of plane specific elastic constants in this region. Certainly, it must be mentioned that the course of $C_{11}$ with temperature for α-iron, given in Reference [7], is also further decreasing for temperatures higher than 700 K. This might contribute to the steeper degression of the lattice plane specific $Young^{\prime }s\;moduli$ and also to a steeper increase of the lattice plane specific $Poisson\;ratio\;\nu^{hkl}$, observed during our in situ X-ray load dilatometry studies as can be seen in Figure 8. A similar behaviour was previously observed for the same diffraction planes in an in situ tensile test experiment of the structural steel S690QL1 by Dutta et al. [20]. In summary it can be stated that up to nearly $400\;{}^{\circ }C$ for all investigated lattice planes there is no significant difference between the experimentally determined values and the linear course of literature based data. For higher temperatures a deviation from this linear trend can be observed, which is most pronounced for the {200} lattice planes and must be taken into account for stress calculation for in situ diffraction studies on ferritic steels at elevated temperatures.

### 3.2. Macroscopic Elastic Constants

**Figure 10:**
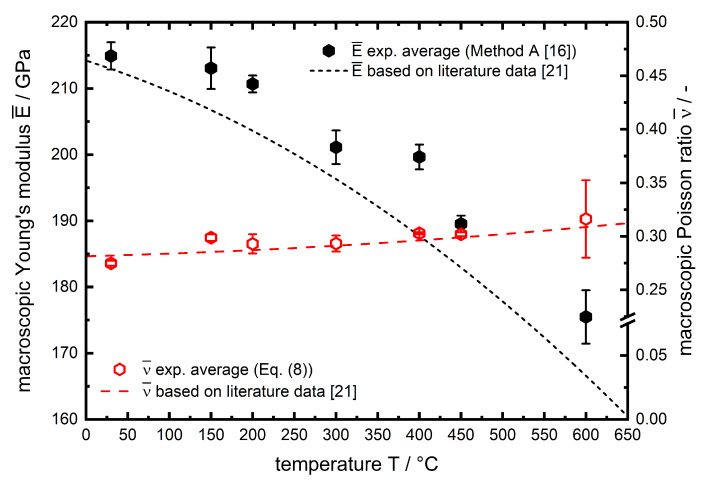
Macroscopic temperature-dependent $Young^{\prime }s\;moduli$ and Poissonratios of AISI 4140 averaged according to Daymond [16]. Literature data is shown as a 3rd order ($\bar{E}$), respectively 2nd order ($\bar{\nu }$) polynomial fit based on data from Miokovic [21].

The results from averaging the lattice plane specific data according to Daymond [16] is given in Figure 10.

Additionally, the temperature course of $\bar{E}$ and $\bar{\nu }$ is shown as dashed lines based on macroscopic data [21] determined by high temperature tensile tests. For both elastic parameters, there is a very good agreement of $\bar{E}$ and $\bar{\nu }$ with the temperature course of conventionally determined (high temperature tensile tests) elastic constants. For all data points the $Young^{\prime }s\;modulus\;E$ is slightly higher compared to literature. These deviations may occur due to chemical fluctuations of the material or simply due to the approach of calculating macroscopic values from lattice specific results versus macroscopic high temperature tensile tests. In this regard it must be mentioned that the approach to use diffraction data to determine temperature dependent elastic constants has a crucial advantage over most macroscopic test methods. The diffraction approach is insensitive to temperature gradients since the data is collected in a localized materials volume. Regarding the current experiment using dilatometry, the induction heating results in temperature gradients along the length of the cylindrical sample. However, this has no impact on the diffraction data presented here. A weak point seems to be the texture of the material, included as $T^{hkl}$ in Equations (6) and (7). In this work, the assumption of of $T^{hkl}=1$ is well justified, since the intensity ratio $I_{max}/I_{min}$ over a single diffraction ring hkl varies between 1.3–2 for all experiments, which allows the conclusion that there is no pronounced crystallographic texture. However, the temperature dependency of the Poissonratio is less pronounced than for the $Young^{\prime }s\;modulus$. The high error bars for $600\;{}^{\circ }C$ can be explained by the lower measurement statistics due to the necessity of applying small compressive loads to ensure purely elastic deformation since the high temperature strength became rather small. All data for the values for $\bar{E}$ and $\bar{\nu }$ determined using of the in situ X-ray load dilatometry approach are tabulated with their corresponding deviations in Table 3.

## 4. Conclusions

Multiple in situ X-ray diffraction dilatometric load experiments were performed for different temperatures and the lattice plane specific temperature-dependent elastic constants for four different hkl of the bcc α-iron phase (ferrite) and for the tbc martenite phase of the hypoeutectoid tempering steel AISI 4140 were determined. The experiments were performed for a temperature range between $30\;{}^{\circ }C$ and $600\;{}^{\circ }C$. From the recorded diffraction data the DECs were determined and the macroscopic elastic constants were averaged using the approach of Daymond [16] from the lattice plane specific results. The complete set of lattice plane specific results for the investigated temperatures is given in Table A1 in the Appendix A. The following conclusions can be drawn:

- In situ X-ray diffraction load dilatometry experiments provide a suitable tool to determine lattice plane specific elastic constants and hence DECs.
- The experiments are much faster in comparison to conventional laboratory X-ray diffraction experiments for determination of DECs; annealing effects for steel samples can be safely neglected.
- Macroscopic $Young^{\prime }s\;moduli$ and Poisson ratios can reliably be derived from averaging multiple hkl specific elastic constants and can further be used as input data to improve FE heat treatment simulations, for example, laser hardening process simulation.
- Only for high-indexed planes with Γ≈0.2, for example, {211}, in the first instance, it can be assumed that the temperature-dependent micro-mechanical behavior can accurately be approximated through the temperature dependency of the macroscopic elastic constants.
- For lower indexed planes hkl like {200} especially above $400\;{}^{\circ }C$ large errors occur through the approximation based on macroscopic elastic constants.
- In summary, for temperatures higher than $400\;{}^{\circ }C$ deviations occur from the thus far observed linear trend of the lattice plane specific $Young^{\prime }s\;moduli$ and Poissonsratios against temperature. These deviations have to be taken into account for precise stress calculations, especially at in situ diffraction studies on ferritic steels at elevated temperatures.

The shown results are useful in two regards. First, high temperature X-ray diffraction stress analysis of AISI 4140 becomes more reliable using the determined temperature-dependent DECs. Secondly, averaging the results delivers a solid data basis of macroscopic elastic parameters which can be used in heat treatment simulations, due to the fail-safe neglect of tempering effects during the fast experimentation.

## Figures and Tables

**Table 1:** Chemical composition of AISI 4140.

C	Si	Mn	Cr	Mo	S	P	Fe
0.43	0.29	0.73	1.00	0.20	0.06	0.02	bal.

**Table 2:** Experimental plan of investigated phases, test temperatures and maximum stress values (tempered after quenching to $30\;{}^{\circ }C$).

Experiment No.	Temperature ${}^{\circ }C$	Max. Stress MPa	Phase
1	30	795	martensite
2	150	795	martensite
3	200	636	ferrite
4	300	795	martensite
5	400	556	ferrite
6	450	795	martensite
7	600	318	ferrite

**Table 3:** Macroscopic temperature-dependent $Young^{\prime }s\;moduli$ and Poissonratios determined from lattice plane specific data based on the approach of Daymond [16] and Equation (8).

Experiment	Temperature	$\bar{E}$	$\bar{\nu }$
No.	${}^{\circ }$C	$10^{3}$ MPa	-
1	30	214.9±2.1	0.275±0.002
2	150	213.3±3.1	0.298±0.003
3	200	210.7±1.3	0.293±0.009
4	300	201.1±2.5	0.293±0.007
5	400	199.7±1.9	0.303±0.001
6	450	189.6±1.2	0.302±0.002
7	600	175.5±4.0	0.316±0.040

## Abbreviations

The following abbreviations are used in this manuscript:

DESY	Deutsches Elektronen Synchrotron
DEC(s)	Diffraction Elastic Constant(s)
HZG	Helmholtz-Zentrum Geesthacht
DCM	Double Crystal Monochromator

## Appendix A

**Table A1:** Lattice plane specific temperature-dependent $Young^{\prime }s\;moduli$, Poissonratios and DECs.

Experiment	Phase	hkl	Temperature	$E^{hkl}$	$\nu^{hkl}$	$s_{1}^{hkl}$	$\frac{1}{2}s_{2}^{hkl}$
No.			${}^{\circ }C$	$10^{3}$ MPa	-	$10^{-6}\;{\mathrm{MPa}}^{-1}$	$10^{-6}\;{\mathrm{MPa}}^{-1}$
1	martensite	{110}	30	217.5±2.4	0.262±0.003	−1.20±0.02	5.80±0.04
2	martensite	{110}	150	214.4±3.8	0.262±0.008	−1.22±0.04	5.88±0.07
3	ferrite	{110}	200	215.3±1.4	0.263±0.007	−1.22±0.03	5.87±0.04
4	martensite	{110}	300	205.7±2.6	0.267±0.007	−1.30±0.04	6.16±0.06
5	ferrite	{110}	400	203.6±1.1	0.271±0.003	−1.33±0.02	6.24±0.02
6	martensite	{110}	450	195.1±1.3	0.279±0.004	−1.40±0.02	6.56±0.03
7	ferrite	{110}	600	178.6±2.4	0.298±0.01	−1.67±0.06	7.27±0.08
1	martensite	{200}	30	166.9±1.8	0.304±0.003	−1.82±0.03	7.81±0.05
2	martensite	{200}	150	164.4±2.3	0.332±0.004	−2.02±0.04	8.10±0.06
3	ferrite	{200}	200	164.3±1.0	0.313±0.008	−1.90±0.05	7.99±0.05
4	martensite	{200}	300	159.2±1.5	0.321±0.009	−2.02±0.06	8.30±0.07
5	ferrite	{200}	400	146.5±1.2	0.322±0.007	−2.20±0.05	9.02±0.06
6	martensite	{200}	450	128.8±2.7	0.324±0.005	−2.52±0.07	10.3±0.12
7	ferrite	{200}	600	105.7±0.6	0.347±0.009	−3.29±0.08	12.7±0.09
1	martensite	{211}	30	218.9±1.7	0.279±0.003	−1.28±0.02	5.84±0.03
2	martensite	{211}	150	216.2±2.3	0.273±0.002	−1.26±0.02	5.89±0.04
3	ferrite	{211}	200	212.9±0.9	0.275±0.002	−1.29±0.01	5.98±0.02
4	martensite	{211}	300	209.4±1.8	0.294±0.006	−1.41±0.03	6.18±0.04
5	ferrite	{211}	400	205.2±0.6	0.305±0.002	−1.49±0.01	6.36±0.02
6	martensite	{211}	450	196.6±1.3	0.295±0.001	−1.50±0.01	6.58±0.02
7	ferrite	{211}	600	183.3±2.2	0.288±0.002	−1.57±0.02	7.03±0.05
1	martensite	{220}	30	217.8±1.7	0.257±0.003	−1.18±0.02	5.77±0.03
2	martensite	{220}	150	216.4±2.4	0.259±0.004	−1.19±0.03	5.81±0.04
3	ferrite	{220}	200	214.7±1.5	0.256±0.008	−1.19±0.04	5.85±0.04
4	martensite	{220}	300	206.4±2.6	0.275±0.006	−1.33±0.03	6.18±0.05
5	ferrite	{220}	400	206.5±1.1	0.281±0.004	−1.36±0.02	6.20±0.03
6	martensite	{220}	450	196.9±1.4	0.281±0.004	−1.43±0.02	6.51±0.03
7	ferrite	{220}	600	178.8±1.0	0.294±0.005	−1.65±0.06	7.23±0.08
